# Icaritin Attenuates HSC Activation by Down-regulating the HIF-1α and TGF-β/Smad Signaling Pathways to Ameliorate Liver Fibrosis

**DOI:** 10.2174/0109298673362768250417052953

**Published:** 2025-05-05

**Authors:** Keping Feng, Qiaoman Fei, Na Huang, Ke Du, Chengbo Zhang, Yudan Fan, Ying Zhou, Yaping Zhao, Pengfei Liu, Zongfang Li

**Affiliations:** 1 National-Local Joint Engineering Research Center of Biodiagnostic & Biotherapy, The Second Affiliated Hospital of Xi'an Jiaotong University, Xi'an, 710004, China;; 2 Clinical Research Center for Hepatic& Splenic Diseases of Shaanxi Province, Xi'an, 710004, China;; 3 Shaanxi International Cooperation Base for Inflammation and Immunity, Xi'an, 710004, China;; 4 Shaanxi Provincial Academician Workstation, The Second Affiliated Hospital of Xi'an Jiaotong University, Xi'an, 710004, China

**Keywords:** Icaritin, hepatic stellate cell (HSC) activation, TGF-β/Smad, HIF-1α, liver fibrosis, antifibrotic therapy

## Abstract

**Introduction:**

Icaritin is a bioactive flavonol isolated from the Chinese medicinal herb *Epimedium*. The comprehensive understanding of antifibrotic effects and associated molecular mechanisms of icaritin remains incomplete. This study aims to explore the protective effects of icaritin against liver fibrosis and to further elucidate the mechanisms involved.

**Methods:**

Human hepatic stellate LX-2 cells stimulated with TGF-β1 and a carbon tetrachloride (CCl_4_)-induced liver fibrosis mouse model were employed. *In vitro* assays were carried out to evaluate collagen type I (COL I) and α-smooth muscle actin (α-SMA) expression, while *in vivo* studies assessed fibrosis alleviation. Molecular mechanisms were explored *via* analysis of TGF-β1, phosphorylated Smad2/3, and HIF-1α protein levels using Western blotting.

**Results:**

Icaritin suppressed TGF-β1-induced COL I and α-SMA expression in LX-2 cells and ameliorated liver fibrosis in CCl_4_-treated mice. Mechanistically, it significantly reduced TGF-β1 levels, inhibited Smad2/3 phosphorylation, and downregulated HIF-1α protein expression in LX-2 cells.

**Conclusion:**

Icaritin attenuated experimental liver fibrosis through the inhibition of the TGF-β/Smad and HIF-1α signaling pathways, highlighting its therapeutic potential for fibrotic liver diseases.

## 1 INTRODUCTION

Liver fibrosis is a common feature of chronic liver diseases worldwide, primarily attributed to viral hepatitis, alcoholism, metabolic factors, and steatohepatitis resulting from prolonged liver injury [1]. Liver fibrosis manifests as an excessive production and accumulation of extracellular matrix (ECM) in response to injury [2]. Hepatic stellate cells (HSCs), the primary mesenchymal cells in the liver and the source of fibrogenic cells, play a crucial role in the development of hepatic fibrosis. Activation of HSCs is triggered by various cytokines and reactive oxygen species released from damaged hepatocytes. This activation process involves HSCs transition from a quiescent state to myofibroblasts, which are responsible for synthesizing key ECM components, such as collagen type I and α-smooth muscle actin (α-SMA) [3, 4]. The activated HSCs are major targets for antifibrotic therapy. Inhibiting or reversing the activation of HSCs can provide an effective clinical treatment of liver fibrosis.

Chinese herbal medicine has a long history in the prevention and treatment of liver fibrosis in China. Icaritin is a small molecular compound derived from the herb *Epimedium*, a traditional Chinese medicine used for nourishing the liver and kidney, and has garnered significant interest due to its broad spectrum of therapeutic effects, particularly in oncology [5, 6]. Accumulating research indicates that icaritin exhibits diverse pharmacological activities, including anti-tumor effects [7]. Numerous preclinical studies and clinical trials have explored the potential therapeutic uses of icaritin across various diseases, including cancer. Recently, a novel drug primarily composed of icaritin was approved for treating advanced liver cancer. However, the mechanistic basis of icaritin's antifibrotic effects remains poorly characterized.

Hypoxia is a significant factor that increases the expression of hypoxia-inducible factor-1α (HIF-1α) and stimulates the activation of hepatic stellate cells (HSCs), contributing to the development of liver fibrosis. Oxygen is crucial for cell survival, and hepatic oxygen deficiency can cause fibrosis [8-10]. Importantly, hypoxia-inducible factor-1α (HIF-1α) expression is upregulated under hypoxic conditions, promoting the activation of HSCs and thereby contributing to liver fibrosis [11, 12]. Recent studies have indicated that hypoxia enhances the activation of the transforming growth factor-β/small mother against decapentaplegic (TGF-β/Smad) pathway and collagen deposition in fibroblasts through the upregulation of HIF-1α [13, 14]. Transforming growth factor β1 (TGF-β1) is closely associated with liver fibrosis and is recognized as one of the most critical fibrotic cytokines identified to date [15, 16]. Once activated, TGF-β binds to a heteromeric complex of type I and type II transmembrane receptors, leading to phosphorylation and activation of receptor-regulated R-Smads (Smad2/3) [17] and then initiating the expression of TGF-β target genes, such as COL1A1 [18].

This suggests that the TGF-β/Smad pathway could be a viable target for HIF-1α and that HIF-1α may contribute to the initiation and progression of liver fibrosis by modulating the TGF-β/Smad pathway. Thus, enhancing the hypoxic microenvironment and regulating the activation of the HIF-1α/TGF-β/Smad pathway to suppress hepatic stellate cell activation represents an effective therapeutic approach for treating liver fibrosis.

Our study aimed to validate the antifibrotic properties of icaritin using the human stellate cell line LX-2 and experimental models of CCl_4_-induced liver fibrosis while also elucidating its underlying mechanisms of action. Our findings indicated that icaritin effectively suppressed HSC activation and halted the progression of liver fibrosis both *in vitro* and *in vivo*. Specifically, icaritin treatment led to reduced expression of HIF-1α and inhibition of the TGF-β/Smad signaling pathway in LX-2 cells. Collectively, these findings suggest that icaritin could be a promising therapeutic option for liver fibrosis.

## 2 MATERIALS AND METHODS

### 2.1 Cell Culture and Treatment

The LX-2 cell line (Cat No: TCH-C391) was obtained from Haixing Biosciences (Suzhou, Jiangsu, China). LX-2 cells were cultured in Dulbecco's Modified Eagle Medium (DMEM) supplemented with 10% (vol/vol) fetal bovine serum (FBS) and 100 U/ml penicillin/streptomycin. The cells were maintained in a humidified incubator with 5% CO_2_ at 37°C. For the experiments, LX-2 cells were pre-incubated with 5 ng/ml TGF-β1 for 6 hours, followed by treatment with or without icaritin (MCE/HY-N0678) for 24 hours before collection.

### 2.2 Cell Viability Assay, Apoptosis Assay, and Wound-Healing Assay

To assess cell viability, the Cell Counting Kit-8 (CCK-8) assay from Beyotime Biotechnology (Shanghai, China) was employed following the manufacturer's instructions. Apoptosis was quantified using the FITC Annexin V Apoptosis Detection Kit from Beyotime Biotechnology. Briefly, LX-2 cells treated with or without icaritin (80 μM) were harvested and suspended in an Annexin-binding buffer. The cells were then incubated with Annexin V-FITC and PI for 15 minutes at room temperature in the dark and then analyzed immediately using a BD FACS Canto II flow cytometer (BD Biosciences) and analyzed with FlowJo software (FlowJo, LLC, Ashland, OR). For the wound-healing assay, equal numbers of LX-2 cells were seeded into six-well plates. Subsequently, cell monolayers were wounded using a pipette tip to create a gap in the plates. After treatment with or without icaritin (80 μM), the migration of fibroblasts into the cleared area was observed under a microscope at specific time points.

### 2.3 Network Pharmacology Analysis

To screen the potential targets of icaritin, the three-dimensional structure of icaritin was retrieved through the PubChem database, and the icaritin was imported into the PharmMapper database to select human species and predict the target of icaritin. The Canonical SMILES number of icaritin was entered into SwissTargetPrediction, with the screening condition set to probability > 0. The predicted target proteins were then validated and standardized using UniProt. The targets were summarized, and duplicate targets and irregular targets were deleted. The relevant gene targets of ‘Hepatic fibrosis’ were searched in DisGeNET (SCOre_GDA ≥ 0.1), GeneCards (relevance score≥1), and OMIM databases. The predicted target genes were combined as disease-related gene data, and duplicate data were deleted. To construct a PPI network, Venny2.1 was used to identify the common target of icaritin and hepatic fibrosis, and a Wayne diagram was drawn. The common target dataset was imported into the STRING database (Homo Sapiens was selected), and the minimum interaction threshold was set as 0.7 to obtain the PPI network. The Composition-Target-Disease (C-T-D) network was constructed and visualized using Cytoscape 3.7.2. Each node in the network represented the active ingredient and the key target gene, respectively, and the edge was used to connect the active ingredient and the key target gene. Nodes connected to the network were represented by degree ≥ 5, and common targets were mined by MCODE cluster analyses. The KEGG analysis of key targets was performed, and the species with *p*< 0.05 and human species were selected, and the top 00 KEGG pathways were mapped.

### 2.4 Western Blotting

LX-2 cells were harvested and lysed with lysis buffer containing 50 mM Tris-HCl (pH 7.4), 1% Nonidet P-40, 0.25% sodium deoxycholate, 150 mM NaCl, 1 mM EDTA, 1 mM PMSF, and 1 μg/mL each of aprotinin, leupeptin, and pepstatin. Equal amounts of protein were loaded onto an SDS-PAGE gel, followed by transfer to a polyvinylidene fluoride membrane. The membrane was then incubated overnight with primary antibodies (α-SMA, Abcam, ab124964; COL1-A2, Proteintech, 66761-1-Ig; HIF-1α, Zenbioscience, 340462; TGF-β1, Abcam, ab215715; Phospho-Smad2+3, Abcam, ab254407; Smad2+3, Abcam, ab202445; and GAPDH, Proteintech, 60004-1) diluted to 1:1000. A horseradish peroxidase-conjugated secondary antibody (CWBIO, diluted 1:4000) was subsequently applied. Proteins of interest were visualized using enhanced chemiluminescence (Amersham, Arlington Heights, IL) and detected with a Fluorescent and Chemiluminescence Gel Imaging System.

### 2.5 Animal Model of Hepatic Fibrosis

In this study, 6- to 8-week-old male C57BL/6 mice were obtained from the Laboratory Animal Center of Xi'an Jiaotong University (Xi'an, China). The body weights of the experimental mice ranged from 21 g to 25 g. Mice of similar weights were divided into two groups (control group, n = 4; icaritin group, n=5; total number, n=9). Liver fibrosis was induced by administering CCl_4_ *via* intraperitoneal injection (reconstituted in olive oil at a ratio of 1:3, at a dose of 2 μL/g body weight) twice weekly for 6 consecutive weeks [19], with or without concurrent treatment of icaritin (1 mg/kg) also *via* intraperitoneal injection. Mice were euthanized 48 hours after the final CCl_4_ injection, and their livers were collected for subsequent experiments. This study adhered to internationally accepted standards for animal research, following the 3Rs principle. The ARRIVE guidelines were employed for reporting experiments involving live animals, promoting ethical research practices. All procedures were conducted in accordance with the Guidelines of the Biomedical Ethics Committee of the Health Science Center of Xi’an Jiaotong University (Approval No. XJTUAE2023-1714), Health Science Center of Xi’an Jiaotong University, China.

### 2.6 Hematoxylin-Eosin and Sirius Red Staining

Hematoxylin-eosin and Sirius red staining were carried out to evaluate tissue injury and collagen deposition, following established protocols [20]. Images were captured from more than five fields per mouse using a polarized light filter and analyzed using Image-Pro Plus 6.0 software. Each treatment group consisted of at least 3 mice.

## 3 RESULTS

### 3.1 Icaritin Decreased the Levels of α-SMA and COL1-A2 in TGF-β1-stimulated LX-2 Cells

**Fig. (1):**
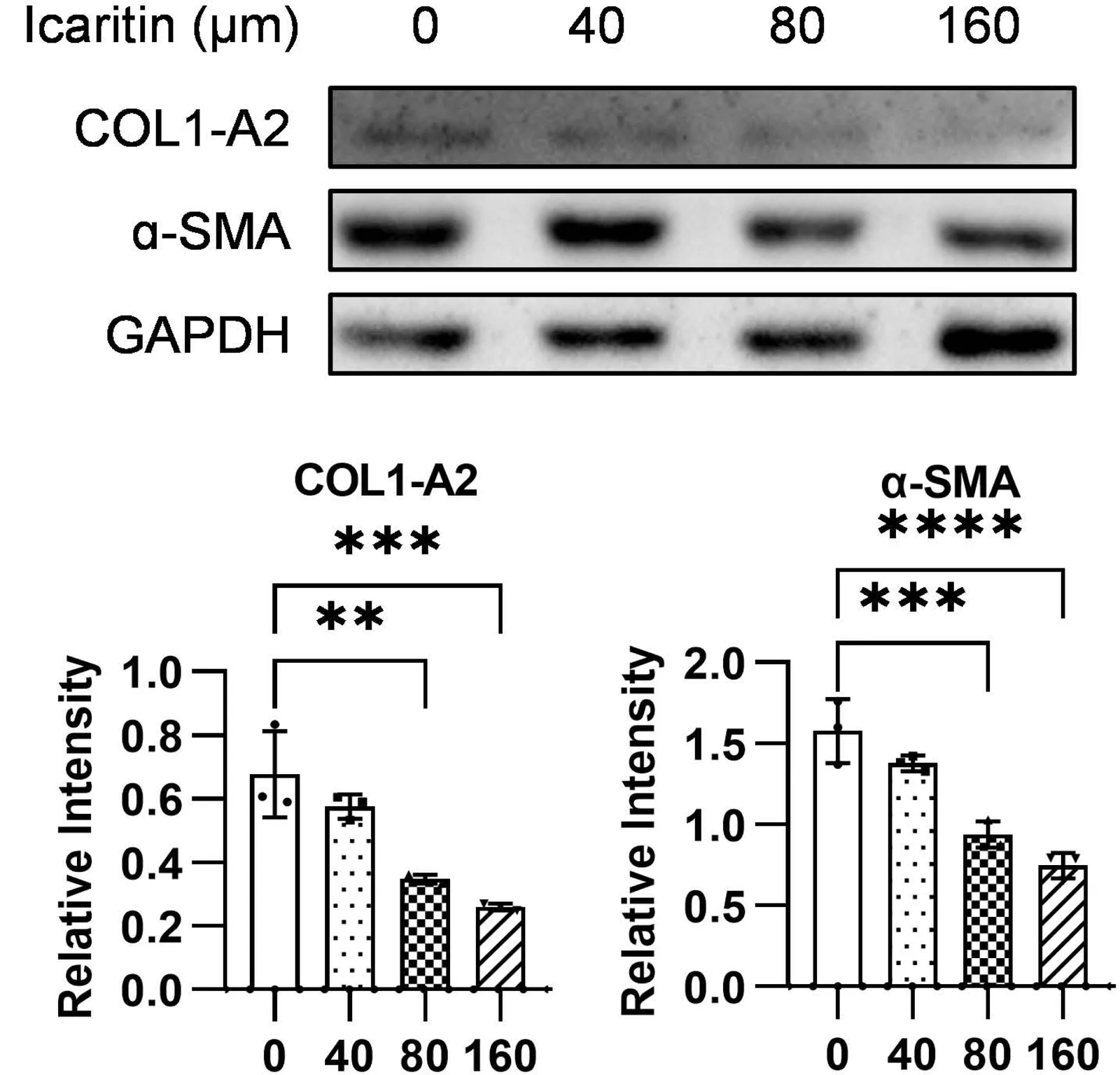
Western blot assays of collagen I-A2 and α-SMA in LX-2 cells treated with icaritin (0 μM, 40 μM, 80 μM, and 160 μM) in the presence of TGF-β1 (5 ng/ml) and the quantitative analysis of the protein expressions (n = 3). All data were presented as means ± SEM. **p < 0.01, ***p <0.001, ****p <0.0001.

Activated HSCs secrete abundant collagen type I (collagen I) and α-SMA, leading to excessive extracellular matrix (ECM) deposition. We investigated whether icaritin could inhibit HSC activation. To explore the mechanisms of icaritin's inhibition of liver fibrosis *in vivo*, we conducted *in vitro* studies using LX-2 cells, a human HSC cell line. TGF-β1 is well-established as a primary activator of HSCs and a critical mediator in the progression of liver fibrosis. LX-2 cells were exposed to 5 ng/ml TGF-β1 for 6 hours, followed by treatment with or without icaritin for 24 h. Western blot analysis revealed that compared to the group stimulated with TGF-β1 alone, the expression levels of α-SMA and COL1-A2 were significantly reduced in a dose-dependent manner in the TGF-β1 + icaritin group (Fig. **1**). These findings indicate that icaritin effectively attenuated TGF-β1-induced activation of HSCs.

### 3.2 Icaritin Suppressed the Migratory Capacity of LX-2 Cells Stimulated by TGF-β1

**Fig. (2):**
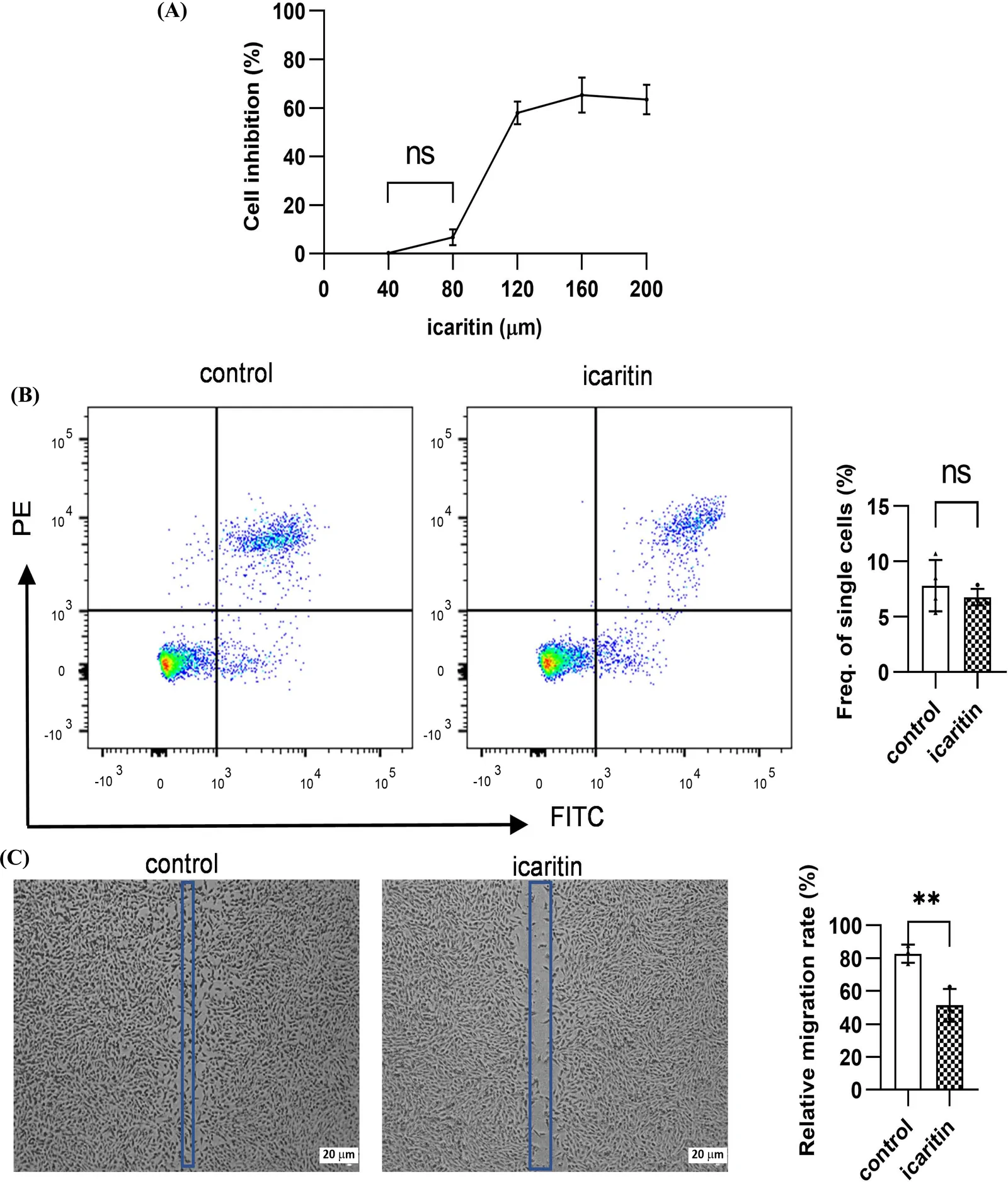
Effect of icaritin on cell proliferation, apoptosis, and migration of LX-2 cells. (**A**) Cell proliferation was determined by CCK8 assay. LX-2 cells plated in 96-well plates were treated with icaritin (40 μM, 80 μM, 120 μM, 160 μM, and 200 μM) for 24 h. (**B**) LX-2 cells were cultured for 24 h, then incubated with icaritin (80 μM) for up to 24 h. Then, cell apoptosis was measured by Annexin V-FITC and PI staining. (**C**) Wound-healing assays revealed that icaritin (80 μM, 24 h) inhibited the migration capacity of fibroblasts. Data are presented as means ± SEM. ** *p* < 0.01; ns, non-significant.

HSC proliferation, apoptosis, and migration are crucial in the progression of fibrosis. To assess the impact of icaritin on the proliferation, apoptosis, and migration of LX-2 cells, LX-2 cells were treated with icaritin at concentrations ranging from 40 μM to 200 μM. The CCK8 assay revealed that icaritin treatment below 80 μM did not affect LX-2 cell proliferation (Fig. **2A**). Next, we evaluated the effect of icaritin on LX-2 cell apoptosis using Annexin V-propidium iodide (PI) staining and flow cytometry. The results indicated that icaritin had minimal impact on the apoptosis of LX-2 cells (Fig. **2B**). Additionally, a wound-healing assay confirmed that icaritin significantly inhibited the migratory capacity of HSCs (Fig. **2C**).

### 3.3 Pharmacological Mechanisms of Icaritin in the Treatment of Liver Fibrosis Analyzed using Network Pharmacology

**Fig. (3):**
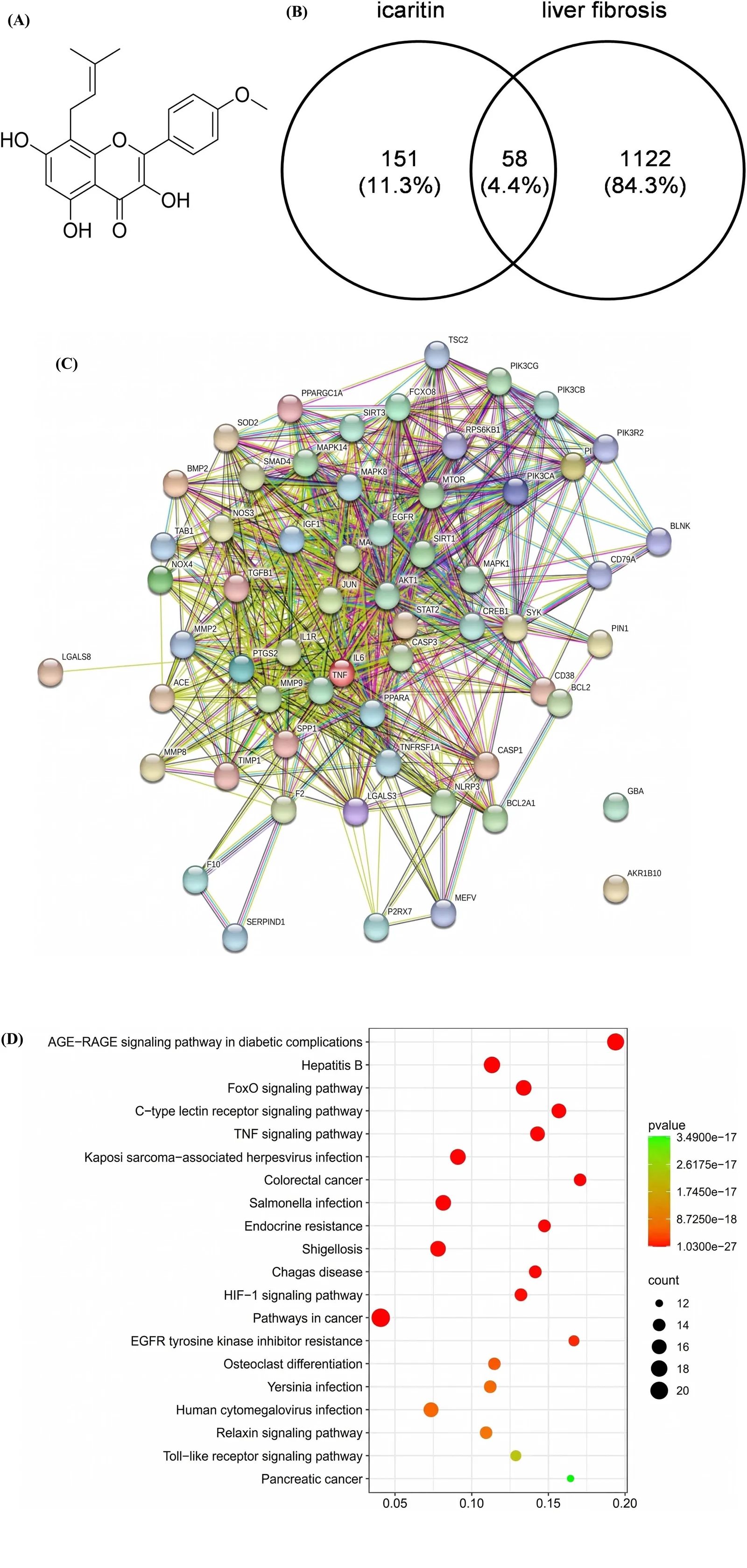
Network pharmacology analysis of icaritin in the treatment of liver fibrosis. (**A**) The chemical structure of icaritin. (**B)** Venn diagram of icaritin targets and liver fibrosis targets. (**C**) Drug-component-disease-target network diagram. (**D**) The top 20 most enriched KEGG pathways were calculated and plotted.

The molecular structure of icaritin is presented in Fig. (**3A**). We conducted detailed research on the targeting and molecular mechanisms of icaritin in liver fibrosis using a multi-omics approach that integrates pharmacogenomics and proteomics. This thorough investigation will enhance our understanding of the therapeutic targets and pathways implicated in the effectiveness of icaritin against liver fibrosis. Utilizing a Venn diagram, we identified 151 potential targets of icaritin and intersected them with 1122 targets associated with liver fibrosis, resulting in a total of 58 intersecting targets (Fig. **3B**). These targets are indicative of icaritin's potential as an anti-liver fibrosis therapy. Fig. (**3C**) illustrates the PPI network of icaritin's targets in anti-liver fibrosis. The KEGG pathway analysis of icaritin's targets in anti-liver fibrosis is depicted in Fig. (**3D**). The main pathways through which icaritin exerts its anti-liver fibrosis effects include the HIF-1 signaling pathway. These pathways underscore the therapeutic potential of icaritin in treating liver fibrosis.

### 3.4 Icaritin Suppressed the HIF-1α Signaling Pathway *in Vitro*

**Fig. (4):**
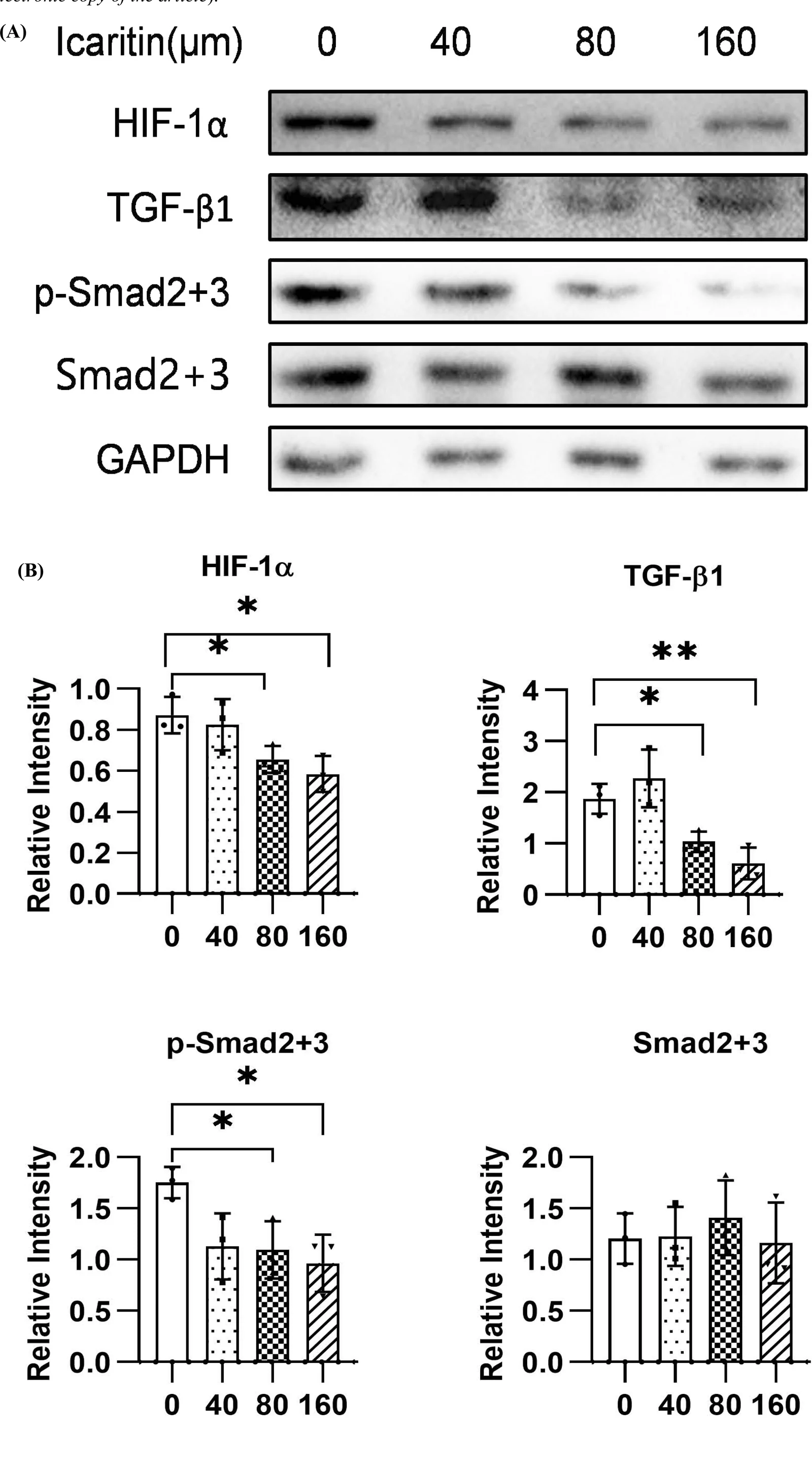
Icaritin down-regulated HIF-1α and TGF-β/ Smad2+3 signaling pathway *in vitro*. (**A**) Western blot analysis for HIF-1α, TGF-β1, p-Smad2+3, and Smad2+3 protein levels in LX-2 cells treated with icaritin (0 μM, 40 μM, 80 μM, and 160 μM) in the presence of TGF-β1 (5 ng/ml). (**B**) Quantitative analysis of the protein expressions (n = 3). All data were presented as means ± SEM. *p < 0.05, **p <0.01.

Various approaches can mitigate liver fibrosis *via* the HIF-1 signaling pathway. HIF-1α may promote the onset and progression of liver fibrosis through its regulation of the TGF-β/Smad pathway. In this study, western blot analysis revealed that the protein levels of HIF-1α in the TGF-β1 + icaritin group were markedly reduced compared to the TGF-β1-stimulated group (Fig. **4**). Collectively, these findings suggest that icaritin inhibited TGF-β1-induced activation of HSCs by downregulating the HIF-1α signaling pathway *in vitro*.

### 3.5 Icaritin Down-regulated TGF-β/Smad Signaling Pathway *in vitro*

The TGF-β1-mediated signaling pathway involves the phosphorylation of Smad 2/3. To delve deeper into the molecular mechanisms underlying the suppression of HSC activation, we examined the impact of icaritin on TGF-β/Smad signaling. As depicted in Figs. (**4A** and **4B**), western blot analysis revealed that the protein levels of TGF-β1 and p-Smad2/3 in the TGF-β1 + icaritin group were significantly reduced compared to the TGF-β1-stimulated group. Overall, these findings indicate that icaritin inhibited TGF-β1-induced activation of HSCs by downregulating the TGF-β/Smad signaling pathway *in vitro*.

### 3.6 Icaritin Mitigates CCl_4_-induced Liver Fibrosis

To further elucidate the protective effects of icaritin in mouse models of liver fibrosis, we assessed collagen accumulation using immunohistochemistry. As depicted in Fig. (**5**), Sirius red and hematoxylin-eosin staining demonstrated that icaritin treatment significantly reduced hepatic collagen deposition compared to the control condition in the liver fibrosis models. These findings indicate that icaritin attenuated hepatic fibrosis *in vivo*.

## 4 DISCUSSION

Liver fibrosis is a common feature of chronic liver diseases worldwide and lacks effective treatment methods. The sustained activation of HSCs is the primary causative factor in liver fibrosis, leading to an imbalance between fibrogenesis and fibrinolysis and resulting in excessive accumulation of ECM [21]. The critical role of preventing HSC activation in the treatment of liver fibrosis is widely recognized.

A growing body of research has highlighted the promising antifibrotic effects of various natural products. Icaritin, a significant component of traditional Chinese medicine *Epimedium*, exhibits diverse biological activities, such as antimicrobial, antimalarial, anti-inflammatory, antitumor, and immunoregulatory effects. Nevertheless, the specific antifibrotic effects of icaritin and the underlying molecular mechanisms have not been fully elucidated [5-7].

In this study, we investigated the potent effects of icaritin on liver fibrosis using a well-established animal model and in HSCs, exploring the underlying mechanisms. The antifibrotic effects of icaritin were substantiated by the following findings: Firstly, icaritin reduced the protein levels of liver fibrogenic markers α-SMA and COL1-A2 *in vitro* and thus inhibited HSC activation. Icaritin reduced HIF-1α expression and inhibited the TGF-β1 and also phosphorylation of Smad2/3. Icaritin treatment in CCl_4_-induced mice led to histological improvements in liver damage and fibrosis, thereby exerting a therapeutic effect on hepatic fibrosis.

Chronic liver injury and inflammatory stimuli induce connective tissue hyperplasia, leading to fibrotic liver remodeling. Hepatocyte swelling, hepatic sinus stenosis, hepatic vasoconstriction, capillary vascularization, perisinusional fibrous collagen deposition, and fibrous septa formation during hepatic fibrosis may cause or aggravate hepatic hypoxia [9, 10]. The hypoxia of liver tissue will further aggravate the degree of liver fibrosis. HIF-1α is an important transcription factor regulating hypoxia response [22-24]. The degree of liver fibrosis development was significantly reduced in mice with HIF-1α gene knockout, suggesting that HIF-1α expression and signal transduction play an important role in liver fibrosis [25].

There are several strategies to mitigate liver fibrosis *via* the HIF-1α signaling pathway. Research indicates that sorafenib induces HSC ferroptosis through HIF-1α/SLC7A11 signaling, thereby reducing liver damage and fibrosis [8]. Similarly, paeoniflorin exerts anti-inflammatory and anti-fibrotic effects in the liver by orchestrating macrophage polarization *via* the NF-kB/HIF-1α pathway [26].

Recent studies have indicated that hypoxia enhances the activation of the transforming growth factor-β1/small mother against decapentaplegic (TGF-β/Smad) pathway and collagen deposition in fibroblasts through the upregulation of HIF-1α [13, 14]. This suggests that the TGF-β/Smad pathway could be a viable target for HIF-1α and that HIF-1α may contribute to the initiation and progression of liver fibrosis by modulating the TGF-β/Smad pathway. Thus, enhancing the hypoxic microenvironment and regulating the activation of the HIF-1α/TGF-β/Smad pathway to suppress hepatic stellate cell activation represents an effective therapeutic approach for treating liver fibrosis.

## CONCLUSION

In summary, our study revealed that icaritin treatment led to reduced HIF-1α expression and inhibition of TGF-β/Smad signaling in LX-2 cells and mitigated liver fibrosis in fibrotic mice. These findings not only highlight the anti-fibrotic properties of icaritin but also provide insights into its mechanism of action in inhibiting HSC activation and liver fibrosis. Therefore, icaritin holds promise as a potential candidate for preventing hepatic fibrosis. However, there is a lack of gene knockdown or overexpression experiments to verify the role of the key targets predicted by network pharmacology, such as HIF-1α, in anti-fibrosis therapy. In this study, only HIF-1α and TGF-β/Smad pathways were verified. The effects of other key targets among the 58 predicted targets were not confirmed by experiments. We anticipate that these limitations will be addressed and expanded upon in our subsequent research.

## Figures and Tables

**Fig. (5):**
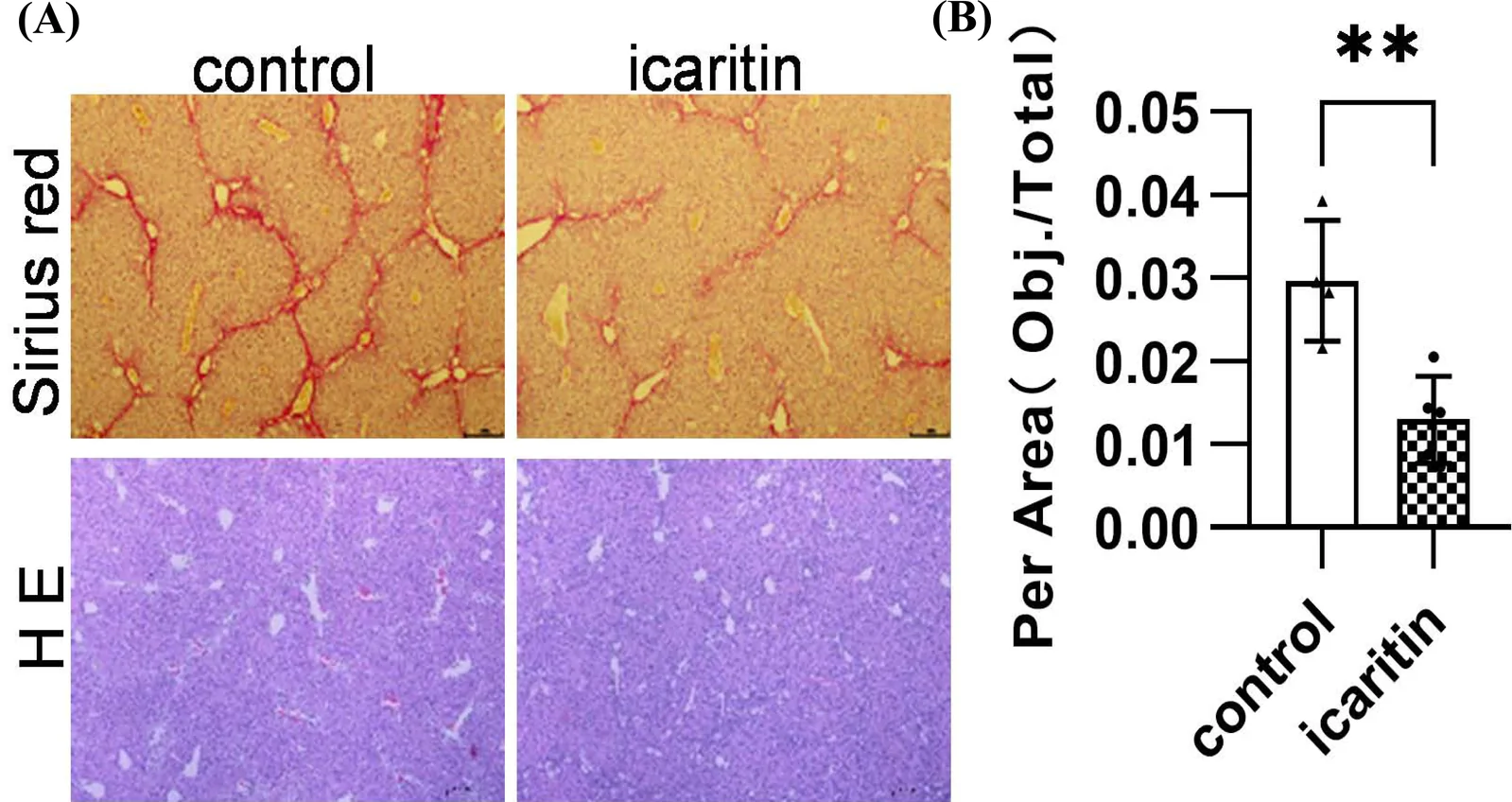
Icaritin ameliorated CCl_4_-induced histological damage and liver injury in liver fibrosis. (**A**) Sirius red staining and hematoxylin-eosin staining. (**B**) Area density analysis of Sirius red staining. Results were shown as the means ± SEM (control group, n = 4, icaritin group, n=5). **p < 0.01.

## Data Availability

All the data and supporting information are provided within the article.
